# Anchorage of Gold Fillings

**Published:** 1893-03

**Authors:** S. B. Palmer


ARTICLE V.

### ANCHORAGE OF GOLD FILLINGS.

BY DR. S. B. PALMER.

The ideal filling is oue of cohesive gold throughout, well contoured and anchored in firm tooth structure. These happy combinations are not always present, thus the requirements must be met by combinations of other preparations. It is true that a few skillful operators claim to accomplish all that is required with cohesive gold alone. Allowing this claim, there is a waste of energy for the operator, and needless strain on the patient without corresponding benefits. One thing should not be lost sight of which is a fixed law in nature: that a metal filling in the mouth exerts an electro potential influence to decompose the fluids that may be between the filling and the walls of the cavity, or in the dentine itself when that is much below normal density; this decomposition is according to the conductivity of the plug and its powers to resist oxidation. Thus gold, being the best conductor and non-oxidizable, acts with greater persistency than tin or amalgam. Let no one be deceived, and make a mistake in trying to circumvent this law by perfect manipulation without a cavity lining which will exclude moisture.

Though cavity lining is not anchorage, it has much to do in laying the foundation for fillings. Your attention is called to a few specific cavities and conditions of dentine, with recommendations for treatment in starting fillings:

*First.* Assuming that an accessible cavity is properly prepared in dentine of normal structure, with undercuts or angles so that the first piece of gold inserted is held firmly, and on which every other piece is made to cohere till the plug is completed, no finer filling can be produced, in appearance or for tooth preservation, and thus credit is given to all who can do this superior work.

Care should be taken not to drill deep anchor pits into the vital dentine, as formerly practiced; much harm has been done by or through the thermal changes thus introduced.

*Second.* Foundations of soft gold for cohesive plugs.

A large portion of gold fillings are done in this manner, and probably each operator has a method which suits him best. The one which best answers my purpose is to commence with one or more cylinders. Without describing the kind of cylinder recommended, we might as well say pellet; there is nothing in the market that answers the purpose, and we may better give a description how to make cylinders now than later on.

Ney's No. 4 foil is best adapted for cylinders, on account of the roughness imparted to it by the book paper under pressure. Smooth foil makes cylinders too hard. The leaf may be cut into half, third, or one-fourth strips, the ribbons folded to a width according to the depth of the cavity. Cylinders should be kept on hand, and the selection made as required. The narrow strips are rolled around a broach, or better, a three-sided point attached to a firm handle; when the roll is drawn from the point, the ends should be slightly pressed with pliers.

Let us now consider a cavity, medium or large, located in the grinding surface of a molar, surrounded by firm enamel borders; bottom of cavity left flat or concave. If the dentine is firm and dense, the cavity is ready for the gold; where soft and sensitive, yet firm enough to warrant filling, varnish the cavity with some quick-drying varnish. I am using Canada balsam dissolved in chloroform. Without waiting for the varnish to harden, line the bottom of the cavity with a piece of gold foil of two or three thicknesses; on this lining place a cylinder which will about fill the orifice of the cavity. If too large, compress; the length of the cylinder may be one-half or two-thirds the depth of the cavity. Then commence with cohesive gold; every piece introduced remains firm and is driven into the folds of the cylinder, condensing and expanding laterally every portion of the plug at the base by lateral pressure against the wall of the cavity, and the anchorage is perfect.

*Third.* Proximal cavities in molars or bicuspids, extending from the cervical border to the grinding surface.

Contour fillings. Matrices well adapted to this class of work are made of rolled aluminum, wide enough to extend from the gums to one-half the length of crown, and beveled to knife edge. Insert the matrix of suitable thickness, and bend the ends away from the cavity, thus giving full view of and access to cavity. The matrix is used only to prevent the first pieces of gold from falling out, and not to mold the filling. The cavity being in readiness, the portion next to the gums only should be varnished, as any such coating on enamel or even dentine prevents the mechanical bite which gold has on enamel without varnish.

Select a cylinder smaller than the cavity, but longer than its depth; carry one end of the cylinder into the cavity, and force the outer end toward the gum on the incline made by the knife-edge matrix. When the cylinder has been firmly pressed to the cervical border of the cavity, another cylinder may be placed by its side, or one on either side, and packed as before; thus a firm and broad foundation is laid, on which cohesive gold may be packed to finish. When done, one end of the aluminum is bent straight, and the piece drawn out by the other end. When the projecting ends of the cylinders are condensed and the plug finished, no portion of the filling will be more perfect than at the cervical border.

*Fourth.* Condition same as the last described, except the cavity extends beneath the gum. A case from practice will illustrate:

A patient, while absent, had occasion to have a bicuspid filled; the cavity extended beneath the gum. The dam was applied, and two unsuccessful attempts made to fill with gold; the patient returned with a phosphate filling. Examination showed it useless to apply the rubber. The cavity was prepared, and filled with amalgam to the cervical border; the remainder was filled with gutta-percha, and the patient dismissed. At the next sitting, the amalgam was cut down for a foundation, the rubber applied, and the tooth filled with gold. One year's time shows no discoloration from the amalgam.

*Fifth.* Cavities on buccal or labial surfaces, extending beneath the gum, making it difficult to apply the rubber, especially the shallow, crescent shaped cavities in centrals and laterals. Prepare the cavity with no more depth than is necessary to remove the softened dentine; let the margin be distinct, and walls at right angles with the bottom of the cavity. Protect the teeth with a napkin or paper; also fill with bibulous paper between the teeth on either sidc of cavity. Dry thoroughly the cavity and gums exposed, and varnish both cavity and gum, allowing the varnish to pass beneath the gum where there is space, which will prevent ingress of moisture by capillary attraction. Line the cavity with crystal gold; first go around the border of cavity, then build across till the cavity is lined with a basket of gold; on this foundation a solid gold plug may be anchored of any cohesive gold without danger of its falling out or of decay around it.

This method is the same as given by Dr. Howard in a clinic at Rochester, and it more than meets his claims in filling the porous dentine, whereby fluids are excluded and secondary decay prevented. With a little practice, filling is made easy with this class of cavities.

Modification and adaptation of the principles here laid down will meet all conditions of anchorage for gold fillings; nor is this preparation of foundations less valuable for amalgam, gutta-percha, or phosphate fillings. Indications for use of varnish under oxyphosphate are sensitive dentine or near approach to the pulp; no acid excitement is experienced with this cavity lining.

Under gutta-percha it is valuable in fastening the filling to the walls of the cavity under conditions unfavorable for exclusion of moisture, as each piece of filling remains fixed in the cavity. It is true chloro-percha and some of the essential oils also cause the filling to adhere, but the lining is much softer and yielding than varnish. In connection with amalgam, it may be used, as with gold as a lining for cavities where poorly calcified dentine must remain as a pulp covering; also in cases where dependence is placed on oxidation to fill the dentine. So far as I can determine, this answers the same with much better appearance. Amalgam fillings inserted in heavy varnish remain bright on the surfaces in contact with the cavity walls which is not the case in many amalgams touching dentine.

Again where there is no action on the metal, there is none on the dentine. Thus amalgam which contains no copper may be used with equally good results, without discoloring the teeth.

In conclusion, science teaches that the result of every operation in filling is the outcome of principles laid down in the foundation and observed to completion. Whatever is success at any time will be for all time, under the same circumstances and conditions.

Progress is based on the knowledge and amplification of principles leading to better methods. Thus we are stimulated in search of more knowledge and higher attainments.
—*Cosmos.*

